# The relation of experience in osteopathic palpation and object identification

**DOI:** 10.1186/2045-709X-21-38

**Published:** 2013-11-12

**Authors:** Rosanna C Sabini, Chadwick S Leo, Alton E Moore

**Affiliations:** 1 Department of Physical Medicine & Rehabilitation, North Shore LIJ - Southside Hospital, 301 East Main Street, Bay Shore, NY 11706, USA; 2 Tioga OB/Gyn Center, 15 Meade Street Suite L1, Wellsboro, PA 16901, USA; 3 C/O MH Northeast Hospital, 18951 N Memor, Humble, TX 77338, USA

**Keywords:** Palpation, Experience, Osteopathy, Manual Medicine

## Abstract

**Background:**

To determine whether osteopathic medical students, fellows, residents, and practicing physicians differ in their ability to identify inanimate objects and if these skills relate to palpatory experience.

**Methods:**

Fifteen commonly known objects were fixed to a board and blinded with a cotton cloth. In Part I of testing, participants were asked to identify 9 objects, with choices provided. In Part II participants were asked to identify 6 objects using one word only. Part III consisted of identifying the shape of an object in Part II.

**Results:**

Eighty-nine osteopathic medical students, fellows, residents, and practicing physicians participated in the study. Overall, correct identification of objects was higher in Part I with choices than in Part II without choices available. No statistically significant difference was found among osteopathic medical students, fellows, residents, and practicing physicians in the correct identification of the objects.

**Conclusions:**

Accuracy in tactile identification of objects among varying levels of palpatory experience was not found. Correlation with clinical palpation cannot be made as it requires a subset of palpatory skills not tested in this study. Accuracy and measurement of palpation should be studied further to demonstrate if palpatory experience improves palpatory accuracy.

## Background

Like other forms of manual medicine osteopathic philosophy requires palpation as a method for diagnosing and treating disease. To obtain these abilities, osteopathic medical schools teach first and second year students the palpatory skills needed to assess and administer osteopathic manipulation in a laboratory setting. Students then spend two years in the clinical setting to refine their skills in taking a complete history, performing a physical examination and improving their palpation.

With continued use of manual medicine, students should improve their palpatory skills and learn to filter insignificant observations [1]. Testing palpation accuracy is challenging because different clinicians vary in their treatment paradigm and professional experience, even though there is standardized medical school training. Moir et al [2] notes that each clinician develops his or her own criteria by which to determine standards of any given test procedure, and there can be a difference in interpretation of the findings [2] and difficulty with these findings being objectively measured [3]. Therefore, it is not surprising that studies showing that palpatory accuracy is related to experience have been conflicting in their findings [2,4-6].

Most articles have failed to show the reliability of palpation in evidence-based clinical practice [7]. Assessing tenderness with palpation can have higher reliability [5,6,8] because patients may contribute to the abnormal findings, whereas identifying anatomical landmarks and other diagnostic tests have been inconsistent [2,4,6,9-13]. Some studies state that the reliability of the palpatory examination has been shown to improve if consensus training is performed prior to testing [5,6,8]. However, a review by Seffinger et al [5] discussed that this was not always demonstrated. Degenhardt et al [7] explained that because patients’ bodies are not static, reliability of palpation can be difficult. The author continued to state that “the neuromusculoskeletal system changes according to the impulses or stresses that an individual experiences and this inherent neurophysiologic variability occurs in both the examiner and subject” [7]. Even if motionless, human tissues respond to touch and change in texture where findings initially diagnosed may no longer be present after multiple examinations via palpation.

Despite the paucity of evidence, professionals continue to use palpation for diagnosis and treatment. Given the difficulties with testing the accuracy of palpatory skills in patients, simplifying the testing conditions to identify objects could help objectively measure haptic skills. Haptic perception is thought to be the perceptual system mediated by active cutaneous and kinesthetic manual exploration [14]. Visual imagery and verbal processing are also proposed theories for identifying the palpatory stimulus [15]. This study sought to investigate the accuracy of object identification using a blinded board as the testing medium of common objects. Haptics, visual imagery and verbal processing of the objects being palpated, processes which are possibly used to palpate clinically, would be assessed. The goal of the study was to show whether osteopathic medical students, fellows, residents, and practicing physicians differ in the accuracy of object identification due to palpatory experience. The statistical null hypothesis was that there would be no difference. No known similar studies have been identified in the literature.

## Methods

**Figure 1:**
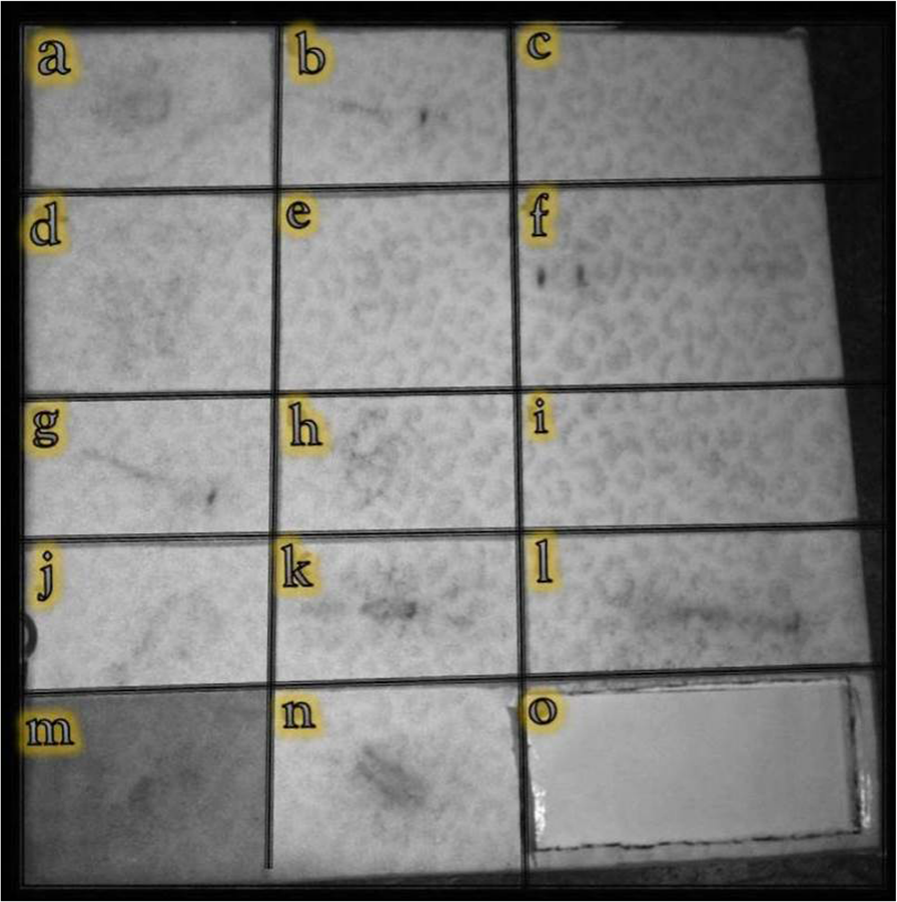
Palpation board covered.

Two identical boards were constructed containing fifteen secured items and covered with a one-eighth of an inch cotton cloth (Figure 1). The objects used are common in day to day activities and were chosen for their similar, yet distinct features. All but one investigator and all participants were blinded to the objects. The unblinded investigator chose the objects tested and constructed the board. The study was exempt by the Institutional Review Board.

Testing was administered during a national osteopathic conference in the exhibit hall. All osteopathic medical students, fellows, residents, and practicing physician attendees were invited to participate. Prior to testing, each participant was given both written and verbal instructions:

– In order to participate, you must agree to the honor code.

– Do not discuss the test with others, regardless if they have or have not taken the test.

– Fill out only one answer sheet.

– Use light but meaningful touch, as too much pressure may damage delicate items.

– All data submitted will be held confidential.

**Figure 2:**
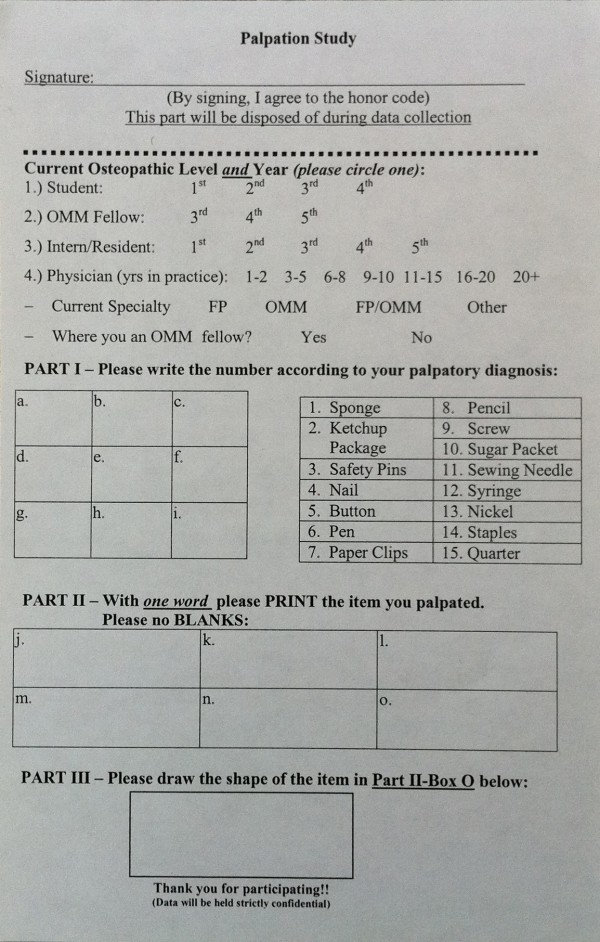
Questionnaire sheet.

Each participant was required to sign their name on the answer sheet (Figure 2), agreeing to the honor code for inclusion in the study. No practice time was given and test taking was limited to 10 minutes to complete.

**Figure 3:**
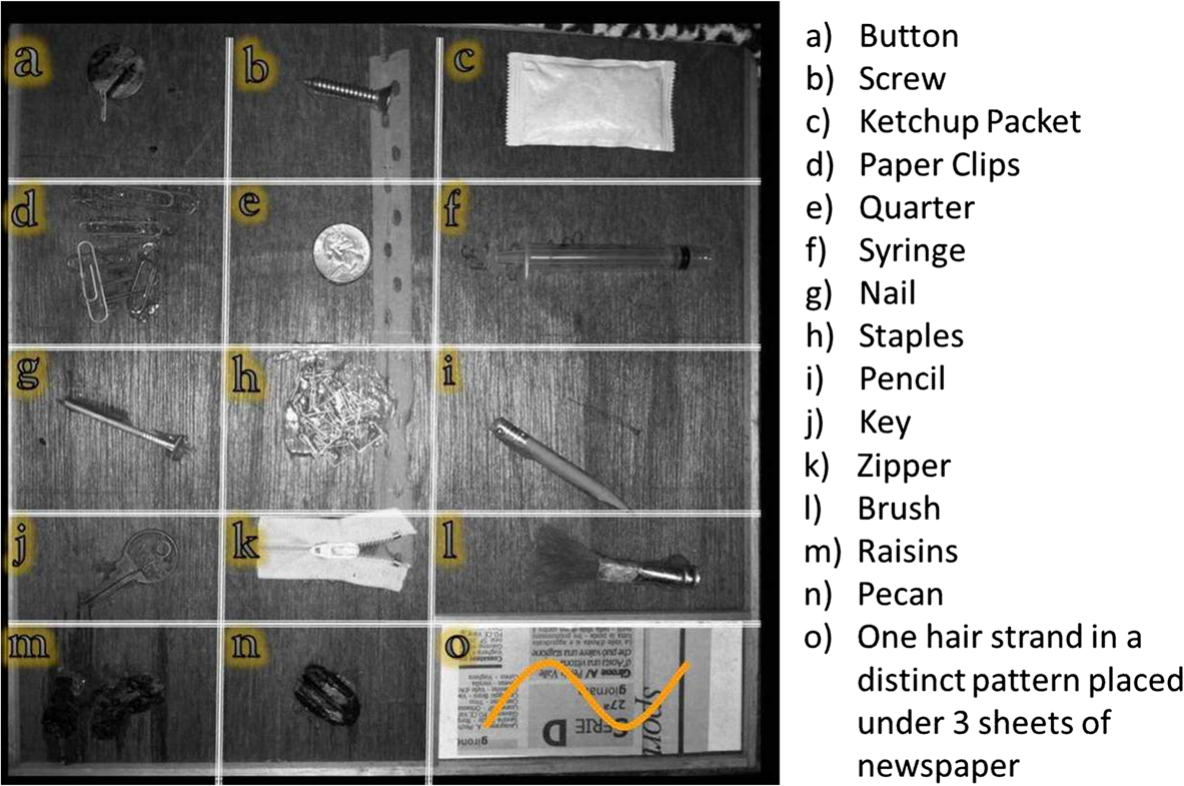
Palpation board uncovered (Part “o” shows graphic shape of item) with item list.

Part I of testing consisted of identifying 9 objects ‘a-i’ and choosing the answers from 15 choices provided. Part II consisted of identifying 6 objects ‘j-o’ and providing one word answers for the object. No acceptable alternate answers were determined prior to the study. Finally, Part III required participants to draw object ‘o’. There was no partial credit given for answers to this item, with the exception of spatial orientation. The uncovered board and item list are shown in Figure 3. Inspection of both boards after completion of the test revealed no alteration from its original form.

After completion, data from answer sheets was converted into an MS Excel Spread sheet. Statistical analysis was conducted using a nonparametric Wilcoxon–Mann–Whitney test, a two-sample rank-sum test. Two-tailed statistical significance was set to a P < 0.05.

## Results and discussion

**Figure 4:**
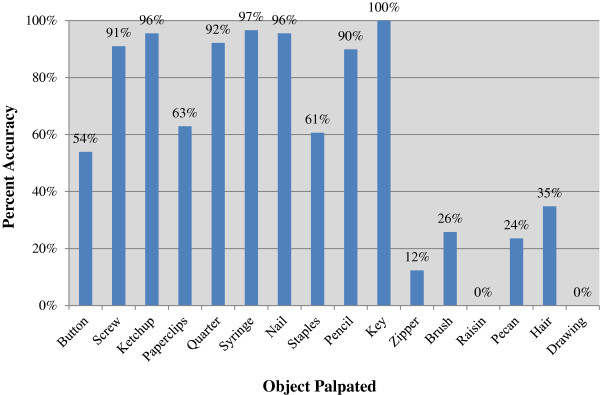
Overall accuracy of each object palpated.

A total of eighty-nine osteopathic medical students (n = 45), fellows (n = 16), residents (n = 6), and practicing physicians (n = 22) participated in the study. The accuracy of identifying the blinded objects was 82% (SD 17.4%) in Part I, 33% (SD 35%) in Part II, and 0% in Part III for all participants. Overall accuracy for each object is shown in Figure 4.

**Figure 5:**
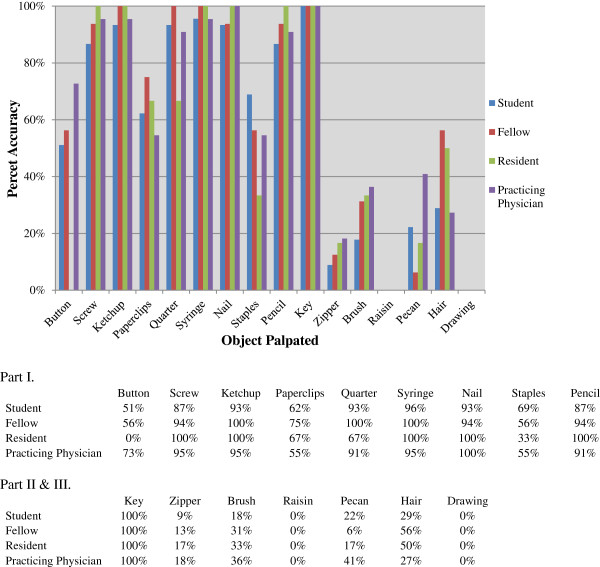
Accuracy of palpation between osteopathic medical students, fellows and practicing physician.

Accuracy of object identification based on osteopathic level of experience is shown in Figure 5. No statistically significant difference was found in the accuracy of object identification among osteopathic medical students, fellows, residents and practicing physicians.

In this study, accuracy of object identification among all participants was higher in Part I than Part II. This was expected because Part I provided choices, whereas Part II required participants to identify the objects without cues. For Part II, because alternate answers were not determined prior to the study, only absolute answers were accepted. Alternate answers chosen post-testing would have biased result interpretation.

Correct identification was unexpectedly low for some objects. In Part I, 54% of participants accurately identified object ‘a’ as a button and 40% incorrectly identified it as a nickel. Yet when evaluating answers for object ‘e,’ 92% of participants correctly identified the item as a quarter. A possible explanation may be that participants may not have reviewed all fifteen choices available prior to beginning Part I. Thus, participants were not mindful of the subtle difference in the objects. For example, the button was the first item and participants may not have identified the holes and incorrectly answered nickel, thereby eliminating nickel as a future choice. If this is in fact the case, the order of objects and resultant elimination of choices becomes an issue.

Insufficient time dedicated to meaningful touch or lack of reexamination may have also been the cause for low accuracy in object identification. For example in Part III, although 35% correctly identified the item as “hair,” 0% of the participants were able to determine how the hair was arranged. In addition in Part I, about two-thirds of the participants correctly identified object ‘d’ as paper clips, while the remaining incorrectly identified the object as safety pins. Although similar, there are characteristics that help distinguish between these two objects. Irrespective of there being a 10 minute time limit, no participant required additional time for testing. It is possible that the amount of time dedicated to each object varied. This leads to the question of how much time should an examiner palpate? With increased palpatory experience, it is assumed that less time is needed.

While a novel study design, affixing objects against a board created a two-dimensional surface, and the cloth blinding the objects may have further impeded tactile abilities. Three-dimensional testing could have improved accuracy if participants freely handled an object in a blinded container. In this experiment, haptic perception was limited by the cloth and is considered to be indirect palpation because the cutaneous feedback has been limited [14]. In addition, accuracy of object identification may have been poor because despite the objects being “common,” participants may not have had intentional experience in handling these objects. Ability to perform this task could possibly rely more on the ability to visually imagine or mentally represent the object and/or the ability to verbally describe the object being palpated [15]. Familiar objects are more likely to be accurately recognized, but even unfamiliar objects are possibly recognized at levels significantly above chance [16]. Therefore, each palpatory experience of an object should improve accuracy with palpations of that object.

Accuracy of object identification may have improved if a training session was performed prior to testing. Conclusions as to whether the results stemmed from pre-testing training would then need to be addressed. Equal amount of training may result in no difference in accuracy of object identification among different levels of osteopathic experience. However, having clinical palpatory experience may generate a measurable difference because the learning curve may be steeper than for someone with less palpatory experience.

Although this study was successful in obtaining a high number of participants in a short period of time, the population was heavily weighted towards osteopathic medical students. Given the study’s setting this may have been unavoidable, because the American Academy of Osteopathy Annual Convocation is attended more by students than interns and residents. On the other hand, testing at such an event was ideal for attracting practitioners who are actively using osteopathic manipulation and are a better population for measuring a difference in tactile skills.

The finding that no statistically significant difference was found between accuracy of object identification and levels of experience is still surprising. However, measuring clinical palpatory accuracy is more difficult to assess. Osteopathic philosophy and teachings focus on palpation of living and moving tissue, a component of palpation which this study did not undertake. In addition, multiple variables are involved in the evolution of one’s clinical palpatory experience and clinicians depend upon a milieu of clinical skill sets learned from experience that is beyond palpation alone. This study was a simple design, and the complexity of palpatory skills evident among varying levels of experience was not measured and contributed to the lack of perceivable difference. However, future studies on measuring palpatory accuracy as it relates to palpatory experience should be performed. Better designed studies could provide light on how training can lead to improved palpatory accuracy.

## Conclusion

Accuracy in tactile identification of objects did not differ among osteopathic medical students, fellows, residents, and practicing physicians. Measuring palpatory skills such as palpation of patients requires a subset of palpatory skills that were not tested in this study. Therefore, accuracy and measurement of palpation needs to be studied further to demonstrate changes with palpatory experience.

## Competing interests

No financial support was received for this manuscript.

All of the authors have no financial disclosure or conflict of interest to report.

## Author’s contributions

All authors conceived the study, participated in its design and coordination and helped to draft the manuscript. All authors read and approved the final manuscript.
